# RNF43 and PWWP2B inhibit cancer cell proliferation and are predictive or prognostic biomarker for FDA-approved drugs in patients with advanced gastric cancer

**DOI:** 10.7150/jca.56014

**Published:** 2021-06-01

**Authors:** Sung-Hwa Sohn, Hee Jung Sul, Bohyun Kim, Hyeong Su Kim, Bum Jun Kim, Hyun Lim, Ho Suk Kang, Jae Seung Soh, Kab Choong Kim, Ji Woong Cho, Jinwon Seo, Youngho Koh, Dae Young Zang

**Affiliations:** 1 Hallym Translational Research Institute, Hallym University Sacred Heart Hospital, Anyang, 14066, Republic of Korea.; 2 Department of Internal Medicine, Hallym University Medical Center, Hallym University College of Medicine, Anyang-si, Gyeonggi-do 14068, Republic of Korea.; 3 Department of Surgery, Hallym University Medical Center, Hallym University Kangnam Sacred Hospital, Singil-ro Yeongdeungpo-gu, Seoul, 07441, Republic of Korea.; 4 Department of Pathology, Hallym University Medical Center, Hallym University College of Medicine, Anyang-si, Gyeonggi-do 14068, Republic of Korea.; 5 Department of Bio-medical Gerontology, Ilsong Institute of Life Sciences, Hallym University, Anyang, Gyeonggi-do, Republic of Korea.

**Keywords:** gastric cancer, PWWP2B, RNF43, docetaxel trihydrate, pelitinib, uprosertib

## Abstract

**Background:** Abnormal regulation of genes has been closely related to gastric cancer. The characterization of gastric cancer has necessitated the development of new therapeutics as well as the identification of prognostic markers to predict the response to novel drugs. In our study, we used RNA sequencing analyses to show that on gastric cancer tissues to identification of gastric cancer prognostic markers. We specifically chose to study RNF43 because it inhibits gastric cancer-related Wnt/β-catenin signaling by interacting with Wnt receptors. PWWP2B was chosen because it is a gene which is downregulated in gastric cancer.

**Methods:** Utilizing RNA sequencing analysis, we evaluated the mRNA expression profile in gastric cancer patients. Also, we used HAP1 cells which is a human near-haploid cell line derived from the male chronic myelogenous leukemia cell line KBM-7. These cell line has one copy of each gene, ensuring the edited allele will not be masked by additional alleles. We investigated the screening of 1,449 FDA-approved drugs in HAP1, HAP1 RNF43 KO and HAP1 PWWP2B KO cells. RNA sequencing data reveals that RNF43 and PWWP2B expression were down-regulated in recurrence gastric cancer patients. Next, we investigated the anti-cancer effects of selected drugs in RNF43 and PWWP2B down-regulated MKN45 gastric cancer cells and xenograft model.

**Results:** Among these FDA-approved drugs, three drugs (docetaxel trihydrate, pelitinib and uprosertib) showed strong inhibitory effects in RNF43 KO cells and PWWP2B KO cells. In MKN45 xenograft model, tumor volumes were significantly reduced in the docetaxel trihydrate, uprosertib or pelitinib-treated group. Our data demonstrated that RNF43 and PWWP2B are a biomarker that predict recurrence of gastric cancer.

**Conclusions:** Our findings suggest that docetaxel trihydrate, uprosertib and pelitinib could be used as novel therapeutic agents for the prevention and treatment of gastric cancer with a decrease in RNF43 and PWWP2B expression.

## Introduction

Gastric cancer (GC) is the fifth most common cancer and third leading cause of cancer deaths worldwide, especially in Eastern Europe, South America, and Eastern Asia (mainly China, Japan, and Korea) 1-4. However, gastric cancer incidence has decreased markedly in Asian countries in recent years 5. In South Korea, despite a decline in incidence, it is the second most common cancer 6. In addition, unsatisfactory treatment outcomes are caused by differences of the molecular basis and intrinsic biological factors. The interplay between poor prognoses related genes such as c-MET, H. *pylori*, the gastric epithelium, and microbiota is among the most critical factors determining the fate of treatment outcome 7-9. In an effort to overcome this problem, and to develop and identify new drug candidates, determining tumor characteristics and treatment parameters is important in Eastern Asia. Furthermore, the majority of cancers are loss-of-function events that defy standard inhibitor-based drug screening strategies. One strategy for overcoming the loss of function is to perform high-throughput screening using target gene knockout (KO) cell lines.

RNF43 is encoded by Wnt target genes, and the loss of expression of this E3 ligase has been predicted to result in hyper-responsiveness to endogenous Wnt signals 10. *RNF43* is a tumor suppressor gene in mucinous ovarian cancers, mucinous pancreatic precancerous cysts, and gastric cancer 11-15. Loss-of-function mutations in RNF43 promotes tumor cell proliferation and result in neoplastic transformation 10. Recent studies revealed that RNF43 suppresses proliferation and induces apoptosis in gastric carcinoma cells 11, 16. The PWWP domain is an essential component of DNMT3B that promotes tumorigenesis and contributes to aberrant DNA methylation in carcinogenesis 17.

In this study, we applied an RNA sequencing (RNA-seq) approach to identify RNF43 (i.e., verify a known marker) and PWWP2B (i.e., explore a novel marker) genes differentially expressed in gastric cancer and adjacent normal tissues from 34 patients. The HAP1 cell line was used to determine how the loss-of-function of RNF43 or PWWP2B correlate with gastric cancer. HAP1 cell line has one copy of each gene, ensuring the edited allele will not be masked by additional alleles. To identify Food and Drug Administration (FDA)-approved drugs that selectively target cancer cells with inactivated RNF43 and PWWP2B genes, we performed a high-throughput screening of 1,449 drugs in HAP1, HAP1 RNF43 KO, and HAP1 PWWP2B KO cells. However, HAP1 cell lines were originally derived from human hematopoetic cells. Therefore, anti-cancer effects of selected drugs were re-tested in *RNF43* and *PWWP2B* down-regulated MKN45 gastric cancer xenograft model. This study has the potential to identify additional genes and drugs involved in GC that may contribute to human GC disease.

## Materials and Methods

### Study subjects and gastric tissue specimen collection

Gastric cancer and adjacent normal tissues obtained from 34 patients with total or subtotal gastrectomy who underwent initial surgery at Hallym University Sacred Heart Hospital from March 2014 to July 2015, were selected as the discovery cohort for RNA-seq. Cases that died within 30 days after surgery were excluded. All cases were prospectively followed up for at least 3 year. **Table 1** summarizes the discovery sets. This study was approved by the Ethics Committee of Hallym University Sacred Heart Hospital (2015-I078). Written informed consent was obtained from all of the participants.

### RNA-seq and differentially expressed gene (DEG) analyses

Gastric cancer and adjacent normal tissues from 34 patients were subjected to RNA-seq. Total RNA was extracted with TRIzol Reagent. Beads containing oligo (dT) were used to isolate poly(A) mRNA from total RNA. mRNA was fragmented, and first-step cDNA was synthesized with random hexamer primers using PCR. Second-step cDNA was synthesized using dNTPs, RNase H, and DNA polymerase I. Next, short double-stranded cDNA fragments were ligated to Illumina sequencing adaptors. DNA fragments were gel-purified and amplified by polymerase chain reaction (PCR). The amplified library was sequenced on an Illumina HiSeq 2500 sequencing machine. The raw reads were saved in the FASTQ format, and the dirty raw reads were removed before analyzing the data. Reads that could be uniquely mapped to a gene were used to calculate gene expression levels, which were measured based on the number of reads per kilobase of transcript per million mapped reads. We identified differentially expressed genes (DEGs) between paired tumor and normal samples and considered *P ≤*0.001 as significant.

### Cell lines and cell culture

The male and female human cell lines used in this study. HAP1, HAP1 RNF43 KO, and HAP1 PWWP2B KO cells (derived from 40year male chronic myelogenous leukemia) were obtained from Horizon. HAP1 KO cell lines were generated using CRISPR-Cas9 gene editing technology in Horizon Genomics (Vienna Austria) as single clones. SNU620 (derived from 59 year female gastric adenocarcinoma), MKN28 (derived from 37 year male gastric tubular adenocarcinoma), MKN45 (derived from 62year female gastric adenocarcinoma), and Kato III (derived from 57year male Signet ring cell gastric adenocarcinoma) cells were obtained from the Korean Cell Line Bank (KCLB, http://cellbank.snu.ac.kr). The cells were grown and maintained under conditions of 100% humidity and 5% CO_2_ at 37°C in Iscove's modified Dulbecco's medium (IMDM) or RPMI 1640 medium supplemented with 10% fetal bovine serum (FBS) and 1% streptomycin and penicillin (Invitrogen Life Technologies, Rockville, MD, USA). The cells were plated onto tissue culture flasks (T-75 cm^2^) at a density of 1 × 10^7^/mL in hormonally defined IMDM or RPMI 1640 medium. The medium was changed every 3 days until the cells reached 80-90% confluence, at which point they were used in the experiments.

### Morphological evaluation

Microscopical studies of the HAP1, HAP1 RNF43 KO, and HAP1 PWWP2B KO cells were carried out with a Microscope. The magnification was 400 fold.

### Cell size analysis

HAP1, HAP1 RNF43 KO, and HAP1 PWWP2B KO cells seeded onto 6-well plates at a density 5 × 10^4^ cells per mL. Cell size was determined using a CytoFLEX flow cytometer (Beckman Coulter, Brea, CA, USA). The use of the flow cytometry parameters forward (FSC) and sideward (SSC) scatter of the cells give an indication on gene KO effects through the relative cell size.

### Quantitative real-time PCR analysis

RNA was isolated from cells using TRIzol reagent (Invitrogen, Carlsbad, CA, USA) according to the manufacturer's instruments, and quantified on a NanoDrop ND-1000 device (Thermo Scientific, Wilmington, DE, USA). Complementary DNA (cDNA) was synthesized using the High Capacity cDNA Reverse Transcription Kit (Applied Biosystems, Foster City, CA, USA). Quantitative real-time (qRT) PCR was performed using Power SYBR Green PCR Master Mix on a LightCycler 96 instrument (Roche Applied Science, Indianapolis, IN, USA). The transcript levels of glyceraldehyde-3-phosphate dehydrogenase (GAPDH) were used for sample normalization. Primer sequences were as follows: RNF43 (FW 5'-CTG TCA CTG GCT AGC AAG G-3'; RW 5'-AGC TTC TCA GCG TCA TTA CC-3'), PWWP2B (RT2 qPCR Primer Assay, Qiagen, Inc., Valencia, CA, USA), and GAPDH (FW 5'-GAG TCA ACG GAT TTG GTC G-3'; RW 5'-TGG AAT CAT ATT GGA ACA TGT AAA C-3').

### Cell proliferation assay

The proliferation of HAP1, HAP1 RNF43 KO, and HAP1 PWWP2B KO cells was assessed using the MTT assay. Cells were cultured in 96-well plates (2 × 10^4^/mL) for 0, 24, 48, and 72 h. MTT solution (5 mg/mL) was added at the end of incubation, which was continued for 4 h. The reaction was terminated by adding a detergent reagent (0.01 M HCl). The absorbance was read at 570 nm using a microplate reader (Synergy 2 Multi-Mode Microplate Reader; BioTek, Winooski, VT, USA).

### Colony forming assay

HAP1, HAP1 RNF43 KO, and HAP1 PWWP2B KO cells were diluted and seeded at a density of approximately 5,000 cells per well in 6-cm dish. After incubation for 7 days, colony formation and growth were visualized with crystal violet staining. After the wells were photographed, the dye was solubilized with methanol and the optical density was measured at 570 nm using a microplate reader.

### Cell migration assay

HAP1, HAP1 RNF43 KO, and HAP1 PWWP2B KO cells were diluted and seeded at a density of approximately 1 × 10^5^ cells per well in 6-well plates or 24-well plates. After incubation for 1 day, a straight scratch was made on the cells using a P200 pipette tip. The cells were then washed with PBS and further cultured with or without docetaxel trihydrate, pelitinib, and uprosertib in IMDM. After incubation for 0, 48, and 72 h, the gap width of the scratch re-population was photographed and then compared with the initial gap size at 0 h.

### High-throughput drug screening

An FDA-approved compound library of 1,448 drugs was purchased from Selleck Chemicals. c-MET inhibitor INC280 was supplied from Novartis (Basel, Switzerland). HAP1, HAP1 RNF43 KO, and HAP1 PWWP2B KO cells were seeded at a density of 2,000 or 3,000 cells per well in 384-well, clear-bottom culture plates with 20 µL IMDM or RPMI 1640 medium containing 10% FBS for 24 h. Then, 10 μM of FDA-approved drug was added to the wells, and the cells were incubated for an additional 48 h. Control cells were not exposed to drugs. On the day of the proliferation assay, the medium was removed, 20 μL of fresh medium was added to each well of the 384-well plates, followed by 5 μL of MTS solution (Cell Titer 96 Aqueous One Solution Cell Proliferation Assay Kit; Promega, Madison, WI, USA), and the plates were incubated at 37 °C for 4 h in a humidified environment with 5% CO_2_. The absorbance was read at 490 nm using a PerkinElmer (Waltham, MA, USA) EnVision luminescence microplate reader. Data were validated using the Zʹ factor analysis. The percentage inhibition was expressed as [viability level of test samples/viability level of control)] × 100.

### Confirmatory growth inhibition assays

The half maximal inhibitory concentrations of the selected drugs of HAP1, HAP1 RNF43 KO, and HAP1 PWWP2B KO cells were measured using the MTS assay for selected drugs at concentrations of 40, 20, 10, 5, 2.5, 1.25, 0.625, and 0.3125 µM for 48 h. On the day of the proliferation assay, medium was removed, and 100 μL of fresh medium was added to each well of 96-well plates, followed by 20 μL of MTS solution, and the plates were incubated at 37 °C for 1 h in a humidified environment with 5% CO_2_. The absorbance was read at 490 nm using a microplate reader (Synergy 2 Multi-Mode Microplate Readers; BioTek, Winooski, VT, USA). The IC_50_ values were determined after fitting growth inhibition curves to dose-response curves using GraphPad Prism software (GraphPad Software Inc., CA, USA).

### Apoptosis analysis

HAP1, HAP1 RNF43 KO, HAP1 PWWP2B KO, and GC (SNU620 cells: high-RNF43, low-PWWP2B; Kato III cells: middle-RNF43 and middle-PWWPB; MKN28: low-RNF43 and high-PWWP2B; and MKN45 cells: low-RNF43 and low-PWWP2B) cells seeded onto 6-well plates at a density 5 × 10^4^ cells per mL were treated with the respective IC50 values of docetaxel trihydrate, pelitinib, and uprosertib (**Table 2**). Cell death was determined using the Annexin V-APC/Propidium Iodide (PI) Apoptosis Detection Kit (Thermo Fisher Scientific, Rockford, IL, USA) on a CytoFLEX flow cytometer (Beckman Coulter, Brea, CA, USA). The percentages of intact and apoptotic cells were calculated using CytExpert software (Beckman Coulter).

### *In vivo* tumor growth inhibition studies

All the experiments and animal handling procedures in this study were approved by the Animal Experimental Ethics Committee of the Asan Medical Center, Seoul, Korea. Six-week-old male BALB/c-nu/nu mice (Joongang Laboratory Animal Inc., Seoul, Korea) were housed in cages, and maintained at 23 ºC with a 12-h light/dark cycle under specific pathogen free conditions. Each mouse was inoculated subcutaneously (s.c.) into the right flank with either 1 × 10^7^ cells/mouse of RNF43 and PWWP2B down-regulated human gastric cancer cell line MKN45. When the average s.c. tumor volume reached 100 mm^3^ (day 0), the mice were randomly divided in the following treatment groups (5 mice per group): vehicle control, docetaxel (positive control, 5 mg/kg/weekly intraperitoneally (i.p.)), pelitinib (10 mg/kg/day orally), and uprosertib (10 mg/kg/day orally). Tumor size was measured twice every week with caliper (calculated volume = shortest diameter^2^ x longest diameter/2). Body weight and tumor size were recorded twice every week. After three weeks, the mice were sacrificed.

### Statistical analysis

The data were statistically analyzed using Prism 5 (GraphPad Software Inc.). All values are presented as the mean ± standard deviation. Statistical significance was determined using one-way ANOV (Bonferroni's Multiple Comparison Test) or Fisher's exact test. A P value < 0.05 indicated statistical significance.

## Results

### Baseline characteristics

A total of 34 subjects were enrolled in this study to gain insight into the molecular pathogenesis of gastric cancer in a Korean population. We searched for genetic alterations using RNA-seq in gastric cancer samples and their matched adjacent normal tissues. By comparing the transcriptome sequences of the cancer tissues with their matched normal tissues, we identified differentially expressed genes, including *RNF43* and *PWWP2B* (**Figure S1**). The associations of *RNF43* and *PWWP2B* expression with clinicopathological characteristics are shown in **Table 1**. The subjects included 19 males (56%) and 15 females (44%), with a median age of 68.6 years (range: 44-87 years). At the 3-year follow-up 11 of 34 patients (32%) had gastric cancer relapse (11 of 11 [100%] in the low RNF43 and low PWWP2B expression group). Low expression of *RNF43* and *PWWP2B* (100%) was significantly associated with recurrence (**Table 1**).

### Cell proliferation and migration regulation by RNF43 and PWWP2B

**Figure 1:**
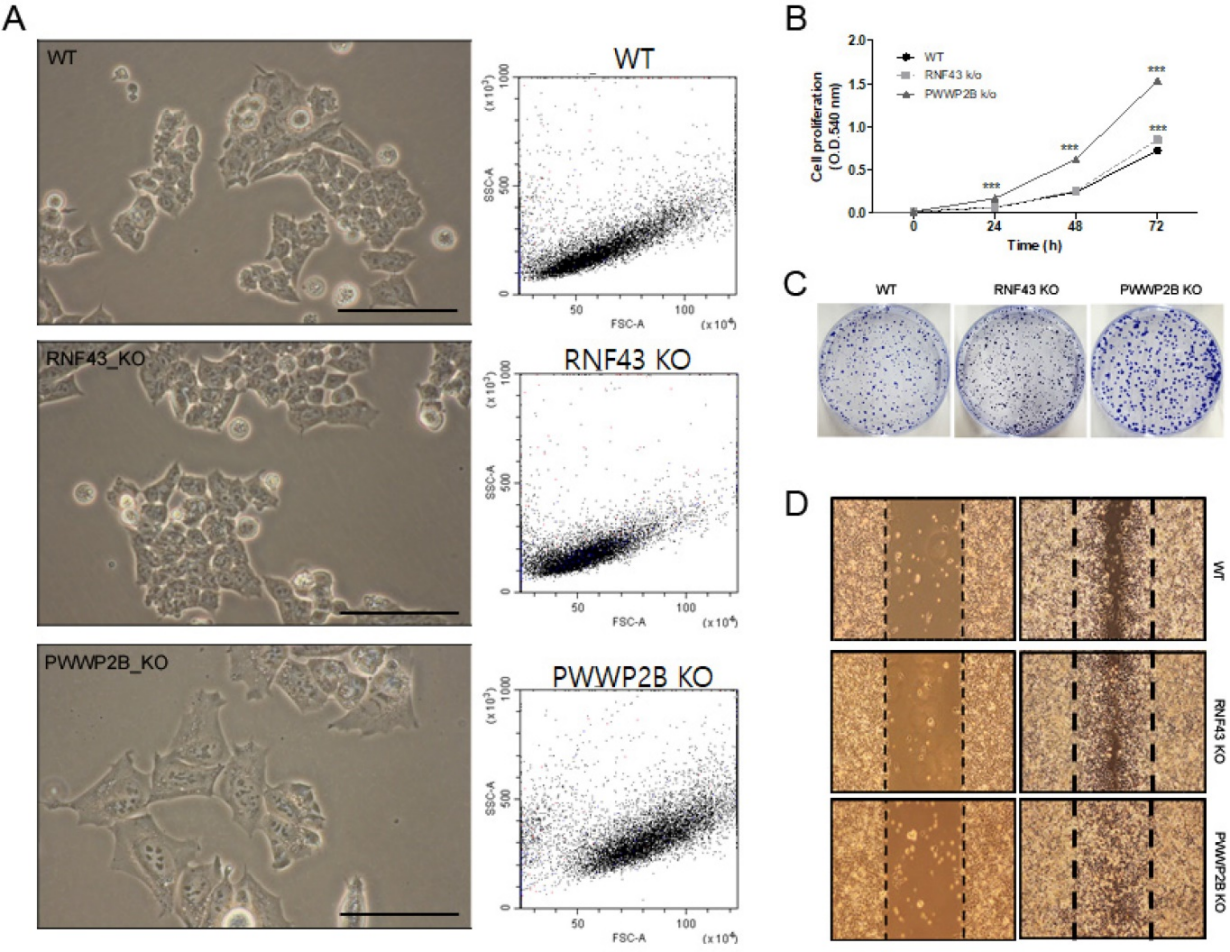
** Characterization of RNF43 KO and PWWP2B KO cells. A** Representative images (left column) and cell size (middle column) of RNF43KO and PWWP2B cells (x400). **B** The MTT cell proliferation assay was performed after 0, 24, 48, and 72 h. Data represent the mean value of three experiments performed in triplicate. **C** The effects of RNF43 and PWWP2B KO on cell proliferation were evaluated by crystal violet staining. **D** The wound-healing assay was used to assess the effects of RNF43 and PWWP2B KO on the migration ability of HAP1 cells. Both RNF43 KO and PWWP2B KO cells showed increased migration ability compared with control cell lines. WT, wild type; KO, knockout; *** *P* < 0.001 compared with the WT.

In light microscopy we first investigated the cell size of *RNF43* KO or *PWWP2B* KO compared to wild type (WT) cells (**Figure 1A**). The KO of *RNF43* and *PWWP2B* show only visible influence on the cell size of *PWWP2B* KO cells. The morphological parameters of the FACS analysis shows only altered cell size in *PWWP2B* KO cells (**Figure 1A**). The effects of *RNF43* KO and *PWWP2B* KO on cell proliferation, we investigated cell proliferation using MTT and colony formation assays (**Figure 1B and 1C**). *RNF43* KO and *PWWP2B* KO increased the proliferation and colony formation of HAP1 cells. Especially, *PWWP2B* KO cell line formed colonies much larger than WT and *RNF43* KO cells, suggesting that the *PWWP2B* KO might endow gastric cancer cells with accelerated proliferative capability. Then we investigated cell migration using the wound-healing assay. *RNF43* KO and *PWWP2B* KO associated with increased migration in HAP1 cells (**Figure 1D**). These results suggest that the loss of *RNF43* and *PWWP2B* may modulate cell migration.

### FDA-approved drug library screen

We to try select effective drugs in recurrence gastric cancer patients with low RNF43 and low PWWP2B expression, by using HAP1 cell line. Those cells have one copy of each gene, ensuring the edited allele will not be masked by additional alleles. To evaluate the effect of the 1,449 FDA-approved drugs on HAP1, HAP1 RNF43 KO, and HAP1 PWWP2B KO cell viability, cells were treated with 10 µM of FDA-approved drugs for 48 h, and the inhibitory effect of the drugs was evaluated using the MTS assay. After 48 h, the cell viability of the drug-treated cells was lower than that of the untreated control cells, with 9 drugs resulting in cell viability less than 40% in HAP1 cells. The cells were treated with different concentrations of each drug for 48 h, and the optimal dose was determined by evaluating cell viability using MTS assays. Treatment with the nine drugs (aprepitant, docetaxel trihydrate, ethinyl estradiol, griseofulvin, INC280, pelitinib, pimobendan, tepotinib, and uprosertib) decreased cell viability in a dose-dependent manner (n = 3). The IC_50_ values of the nine drugs were determined using non-linear regression analysis (**Table 2**). Among these drugs, docetaxel trihydrate, pelitinib, and uprosertib showed the best inhibition rates.

### Effects of three drugs on cell migration

**Figure 2:**
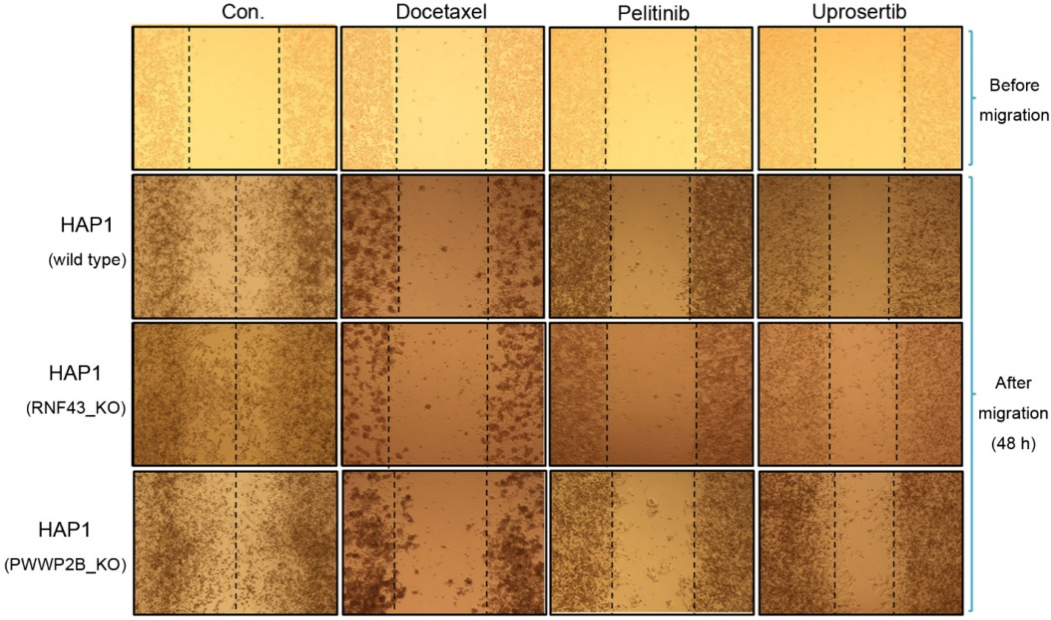
** Anti-migration activities of docetaxel, pelitinib, and uprosertib in HAP1, HAP1 RNF43 KO, and HAP1 PWWP2B KO cells.** The wound-healing assay of HAP1, HAP1 RNF43 KO, and HAP1 PWWP2B KO cells treated with the respective half maximal inhibitory concentration (IC50) values of docetaxel trihydrate, pelitinib or uprosertib. Docetaxel trihydrate-, pelitinib- and uprosertib-treated cells showed inhibited migration ability compared with control cell lines. Experiments were repeated three times. WT, wild type; Con, control; KO, knockout.

To determine the inhibitory effects of docetaxel trihydrate, pelitinib, and uprosertib on HAP1, HAP1 RNF43 KO, and HAP1 PWWP2B KO cells, cell migration was examined by performing a wound healing assay with the respective IC_50_ values of the three drugs (**Figure 2A-C**). The wound gaps in the cells treated with each of the three drugs were significantly wider than those of the untreated groups at 48 and 72 h. IC_50_ values of these drugs, pelitinib and uprosertib showed the best inhibitory effect.

### Effects of three drugs on cell apoptosis

**Figure 3:**
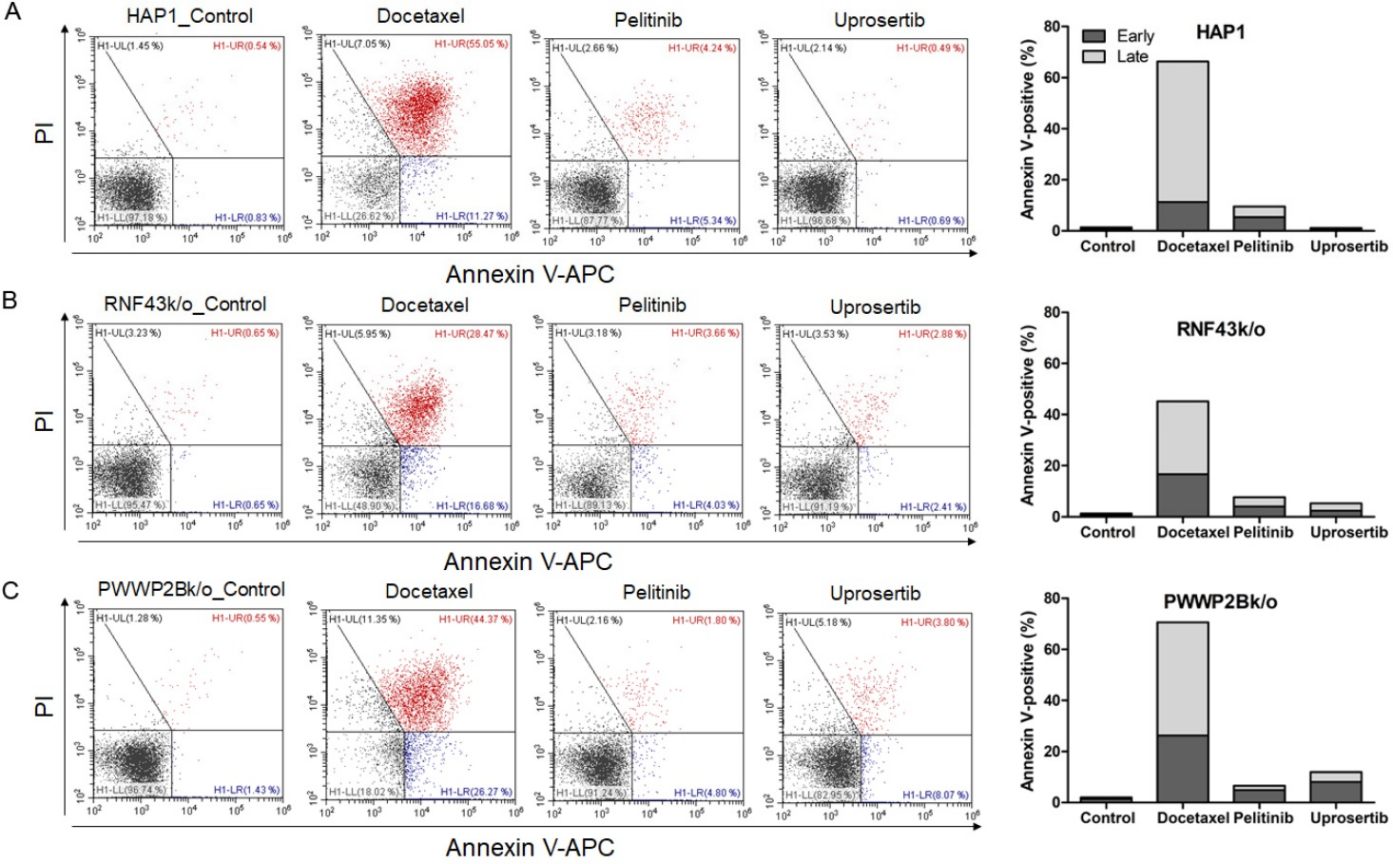
** Apoptotic activities of docetaxel, pelitinib and uprosertib in HAP1, HAP1 RNF43 KO and HAP1 PWWP2B KO cells.** Flow cytometric assay of **A**) HAP1, **B**) HAP1 RNF43 KO, and **C**) HAP1 PWWP2B KO cells treated with the respective half maximal inhibitory concentration (IC50) values of docetaxel trihydrate, pelitinib or uprosertib. Docetaxel trihydrate-, pelitinib- and uprosertib-treated cells showed induced apoptosis ability compared with control cell lines. Experiments were repeated three times. PI, propidium iodide; Con, control; KO, knockout.

**Figure 4:**
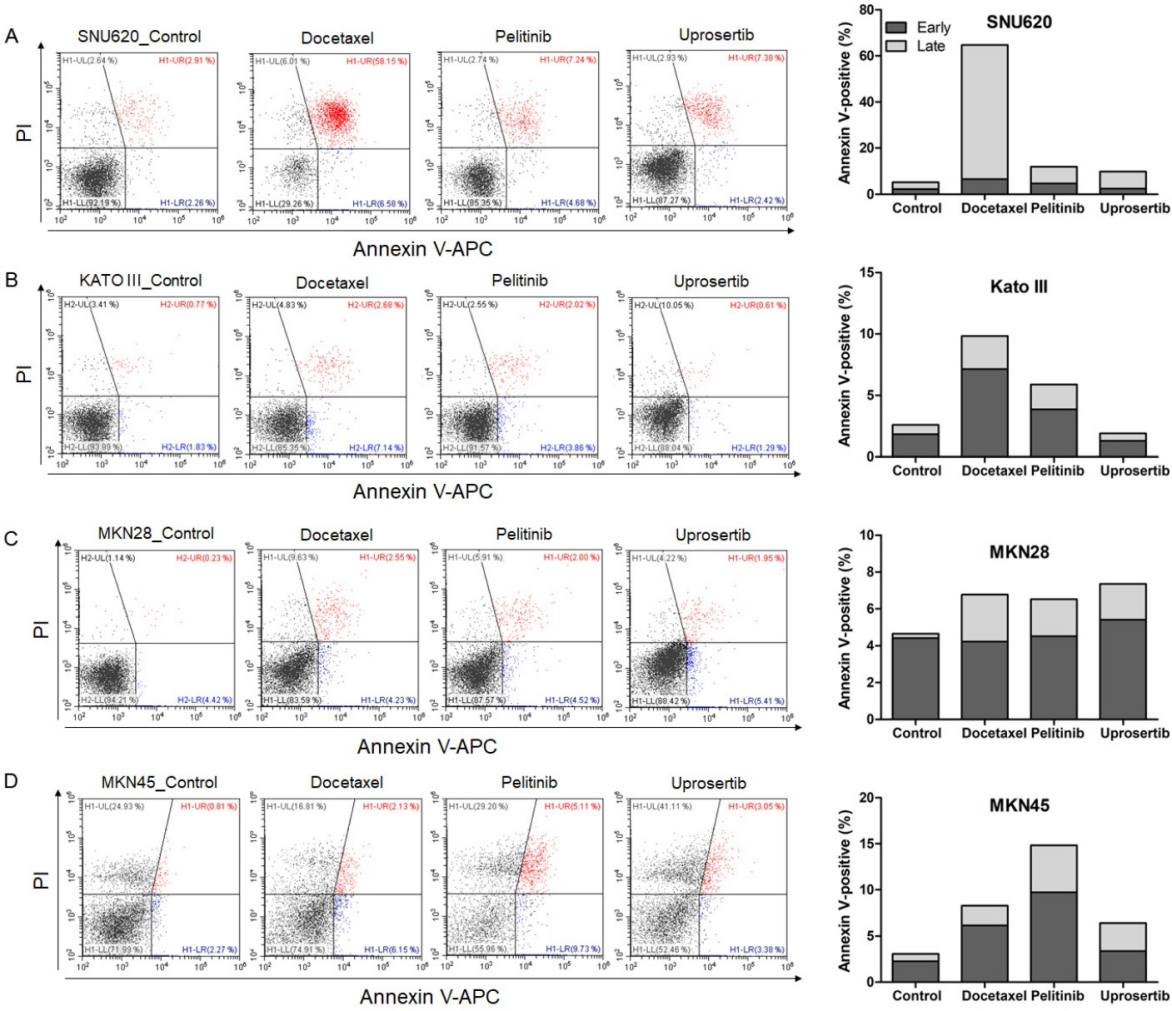
** Apoptotic activities of docetaxel, pelitinib, and uprosertib in gastric cancer cell lines established from metastasis to the peritoneal cavity (Kato III and SNU620 cells) and gastric carcinoma cell lines (MKN28 and MKN45).** Flow cytometric assay of **A**) SNU620, **B**) Kato III, **C**) MKN28, and **D**) MKN45 cells treated with 12nM, 5uM, and 5uM concentration values of docetaxel trihydrate, pelitinib or uprosertib. Docetaxel trihydrate-, pelitinib- and uprosertib-treated cells showed induced apoptosis ability compared with control cell lines. Experiments were repeated three times. PI, propidium iodide; Con, control; KO, knockout.

To evaluate the effects of docetaxel trihydrate, pelitinib, and uprosertib on cell death in HAP1, HAP1 RNF43 KO, HAP1 PWWP2B KO cells, apoptosis was examined by flow cytometry (**Figure 3**). However, these cell lines were leukemia cell line. Therefore, we evaluate the apoptosis effects on GC cells (SNU620 cells: high-RNF43, low-PWWP2B; Kato III cells: middle-RNF43 and middle-PWWPB; MKN28: low-RNF43 and high-PWWP2B; and MKN45 cells: low-RNF43 and low-PWWP2B) was examined (**Figure 4**). Cells were to assess early apoptosis and the rate of apoptosis in a cell population. Docetaxel trihydrate showed the significantly induced cell death rates in HAP1, RNF43 KO, PWWP2B KO, SNU620, and Kato III cells (**Figure 3-4**). Pelitinib showed the best cell death rates in MKN45 cells (**Figure 4D**). *RNF43* and *PWWP2B* genes were weakly expressed in MKN45 cells compared with the other gastric cancer cell types (**Figure S2**). The percentage of apoptotic cells was 10%, 15%, and 6% after exposure to docetaxel trihydrate, pelitinib, and uprosertib, respectively, while that of control cells was only 3% (**Figure 4**). In contrast, these drugs were not effects MKN28 cells, which highly express *PWWP2B* (**Figure 4 and Figure S2**).

### *In vivo* anti-tumor efficacy of three drugs in tumor xenografts

**Figure 5:**
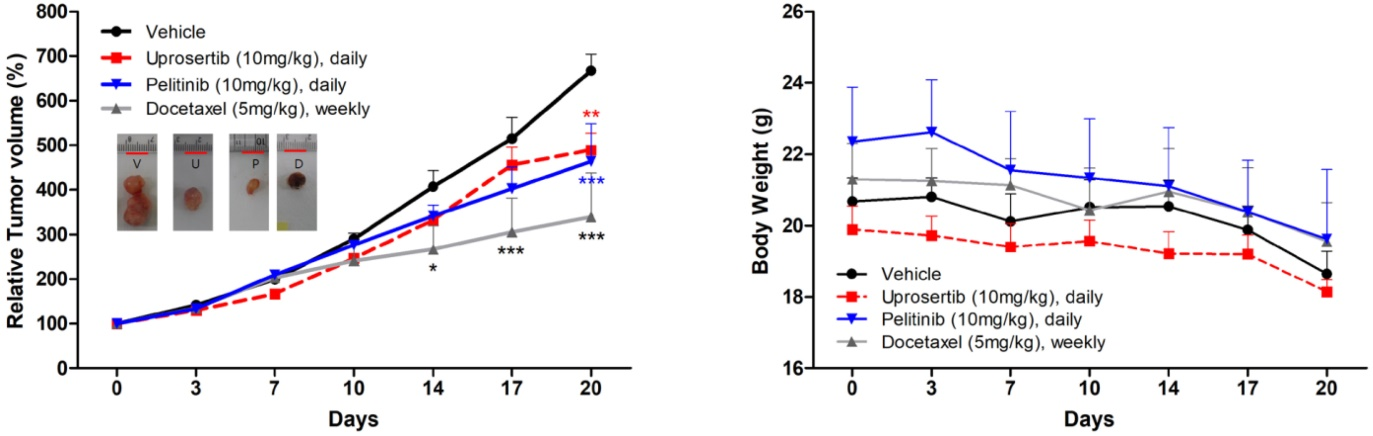
** Anti-tumor growth activities of docetaxel, pelitinib, and uprosertib *in vivo*.** The effects of relative tumor volume and body weight of MKN45 xenograft mouse treatmented with docetaxel trihydrate (D; 5mg/kg/weekly, intraperitoneally), pelitinib (P; 10mg/kg/day, oral gavage) or uprosertib (U; 10mg/kg/day, oral gavage) at described concentrations. All error bars are SD. * *P* < 0.05, ** *P* < 0.01, and *** *P* < 0.001 compared with control (V; vehicle) group.

Prompted by the *in vitro* data supporting a potential anti-tumor activity of docetaxel trihydrate, pelitinib, and uprosertib, we examined the *in vivo* efficacy of 3 drugs on the growth of MKN45 xenograft models. As demonstrated in **Figure 5**, mice bearing s.c. MKN45 tumors were treated with docetaxel trihydrate (blocking tubulin), pelitinib (blocking EGFR), and uprosertib (blocking AKT). The docetaxel trihydrate, pelitinib, and uprosertib could significantly inhibit tumor growth at 3 weeks with the inhibition rate 49%, 31%, and 27% in KMN45 xenografts, respectively. The docetaxel trihydrate and pelitinib were well tolerated as demonstrated by the weight gain of treatment groups over the treatment period (**Figure 5**).

## Discussion

Unsatisfactory treatment outcomes occur in Asian countries due to differences in the intrinsic biological factors and rate of diagnosis of gastric cancer between Western and Eastern countries, which represents a major impediment in this field. Therefore, in the present study, 1,449 FDA-approved drugs were screened according to the tumor characteristic status in Korea.

Aberrant regulation of Wnt/β-catenin signaling is observed in colon, ovarian, lung, prostate, liver, breast, and gastric cancers 16, 18-22. Wnt/β-catenin signaling mediates the epithelial-to-mesenchymal transition in gastric cancer, a process whereby epithelial cells are converted into migratory and invasive cells 23, 24. RNF43 encodes a transmembrane ubiquitin E3 ligase and is a tumor-suppressing gene that suppresses the Wnt/β-catenin signaling pathway, which is intimately involved in the etiopathogenesis of several cancers 11, 25, 26. Using a gastric cancer cohort, we retrospectively evaluated the relationship between *RNF43* and *PWWP2B* expression levels and clinical characteristics (**Table 1**). We found that gastric cancer recurrence patients showed downregulated *RNF43* and *PWWP2B* (100%). In addition, we found *RNF43* and *PWWP2B* mutations in 7 and 6 of 34 gastric cancer patients, respectively. In addition, 34 gastric cancer patients carried germline *RNF43* mutations. The *RNF43* and *PWWP2B* genes are potential biomarkers candidates for patients with advanced gastric cancer. As far as we know, the *RNF43* gene is well known to be associated with gastric cancer, but the *PWWP2B* gene has not been found to be associated with gastric cancer. Therefore, we examined the action of *RNF43* and *PWWP2B* in RNF43 KO and PWWP2B KO cell lines. Our results showed that the cellular capacity for proliferation and migration was markedly increased in RNF43 KO and PWWP2B KO cell lines compared with the wild type control. These data suggest that downregulation of RNF43 and PWWP2B might promote proliferation of gastric cancer cells and could be a condition for the conversion of normal gastric epithelial cells into cancerous cells.

Used simultaneously, cell- and target-based screening procedures might be the optimal methods for promoting cancer drug discovery. They can be used to screen large numbers of drugs to determine their therapeutic potential, and correlations between target genes and clinical characteristics. Therefore, in this study, 1,449 FDA-approved drugs were screened to determine whether they could be used as therapeutic agents for the treatment of gastric cancer using growth inhibition assays of HAP1, HAP1 RNF43 KO, and HAP1 PWWP2B KO cells. Among the 1,449 FDA-approved drugs tested, nine (aprepitant, docetaxel trihydrate, ethinyl estradiol, griseofulvin, INC280, pelitinib, pimobendan, tepotinib, and uprosertib) showed high inhibitory activity; therefore, these drugs were selected for further study. **Table 2** presents the IC_50_ values and effective doses of these drugs. Consistently, we have observed the anti-cancer effects using the docetaxel trihydrate, pelitinib, and uprosertib in concentrations as low as 17nmol/L-3 umol/L for *in vitro* and 5-10 mg/kg for *in vivo* studies. Docetaxel trihydrate, pelitinib, and uprosertib, which showed the highest inhibition and apoptotic rates of the tested drugs, have been shown to have therapeutic utility. For example, AKT signaling is responsible for development of resistance in cancer to various chemotherapeutics drugs 27. The downstream protein GSK3ß is major AKT substrate. Docetaxel trihydrate is used widely to treat recurrent or gastric cancer 28. Docetaxel treatment inhibit phosphorylation state of GSK3ß 29. Pelitinib is used to treat lung cancer 30. Pelitinib is also an inhibitor of epidermal growth factor receptor (EGFR), making it active against putative EGFR-dependent tumor types 31, 32. In addition, Pelitinib inhibit EGF-induced activation of AKT and ERK1/2 in cancer cells 33. Uprosertib is used in recurrent or persistent ovarian cancer, endometrial cancer, and melanoma 34. Uprosertib is a broad AKT inhibitor used not only for the treatment of gastric cancer, but also for AKT-dependent cancers 35. It is well-known that active AKT phosphorylates Gsk3β on serine 9, thereby inactivating it 36. Gsk3β inhibits Wnt/β-catenin signaling by inhibiting AKT in gastric cancer.

## Conclusions

In summary, the results of this study indicate that *RNF43* and *PWWP2B* are downregulation in gastric cancers compared with normal adjacent gastric mucosa. From a clinical aspect, low RNF43 or PWWP2 gene expression showed worse prognosis in overall survival than high gene expression, respectively (http://www.stomachcancerdb.org/). Furthermore, a correlation between low *RNF43* and *PWWP2B* expression and tumor recurrence was seen. Importantly, based on our preclinical finding, docetaxel trihydrate, pelitinib, and uprosertib have significant anti-cancer effects in cancers with low RNF43 and PWWP2B expression (HAP1 RNF43 KO, HAP1 PWWP2B KO cells, MKN45 cells, and KMN45 xenografts). Therefore, further studies are needed to elucidate their mechanisms of action should to aid in the discovery of new therapeutic agents for the treatment of gastric cancer.

## Supplementary Material

Supplementary figures.

## Figures and Tables

**Table 1:** Association of RNF43 and PWWP2B expression with clinicopathological characteristics in 34 gastric cancer patients

Characteristics	RNF43 low/PWWP2B low (%)	RNF43 high^1^/PWWP2B low (%)
Age	69.0 (44-87)	62.5 (61-64)
**Sex**		
Male	17(50.0)	2(5.9)
Female	15(44.1)	0
**Tumor location**		
Pylorus	1 (2.9)	0
Antrum	20 (58.8)	2 (5.9)
Body	6 (17.7)	0
Pylorus ~ Antrum	1 (2.9)	0
Antrum ~ Body	4 (11.8)	0
**Lauren's classification**		
Diffuse	15 (44.1)	0
Intestinal	6 (17.7)	1 (2.9)
Mixed	11 (32.4)	1 (2.9)
**Cancer stage (TNM class)**		
I	1 (2.9)	0
II	9 (26.5)	0
III	22 (64.7)	0
IV	0	2 (5.9)
**Recurrence**		
Yes	11 (32.3)	0
No	21 (61.8)	2 (5.9)

^1^High, fold change >2.0.

**Table 2:** IC_50_ of selected drugs in HAP1, HAP1 RNF43 KO and HAP1 PWWP2B KO cells

Drug	HAP-1	PWWP2B	RNF43
	IC_50_ (uM)		
Aprepitant	17.25	14.89	16.25
Docetaxel Trihydrate (nM)	19.83	19.31	17.19
Ethinyl Estradiol	18.85	15.88	16.42
Griseofulvin	16.00	13.46	15.56
INC280	39.13	37.56	28.08
Pelitinib	3.14	2.03	0.71
Pimobendan	13.23	11.81	12.38
Tepotinib	23.17	21.38	19.07
Uprosertib	4.93	3.02	2.99

## Abbreviations

RNA-seq: RNA sequencing
FDA: Food and Drug Administration
DEG: differentially expressed gene
SNP: single-nucleotide polymorphism
indels: insertions/deletions
IMDM: Iscove's modified Dulbecco's medium
FBS: fetal bovine serum
IHC: Immunohistochemical
IC_50_: half maximal inhibitory concentration
