# Supplementary figures and images for: RNF43 and PWWP2B inhibit cancer cell proliferation and are predictive or prognostic biomarker for FDA-approved drugs in patients with advanced gastric cancer
Source: J Cancer. 2021 Jun 1;12(15):4616–25. doi: 10.7150/jca.56014 (PMC8210561; doi:10.7150/jca.56014)

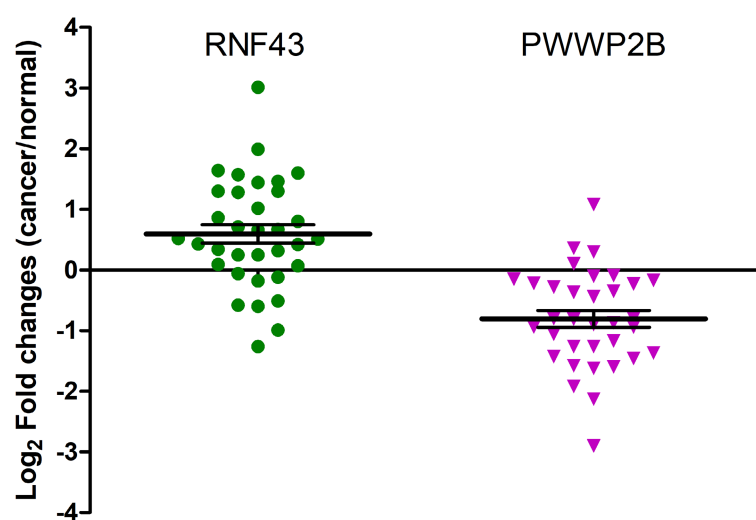

Figure S1.

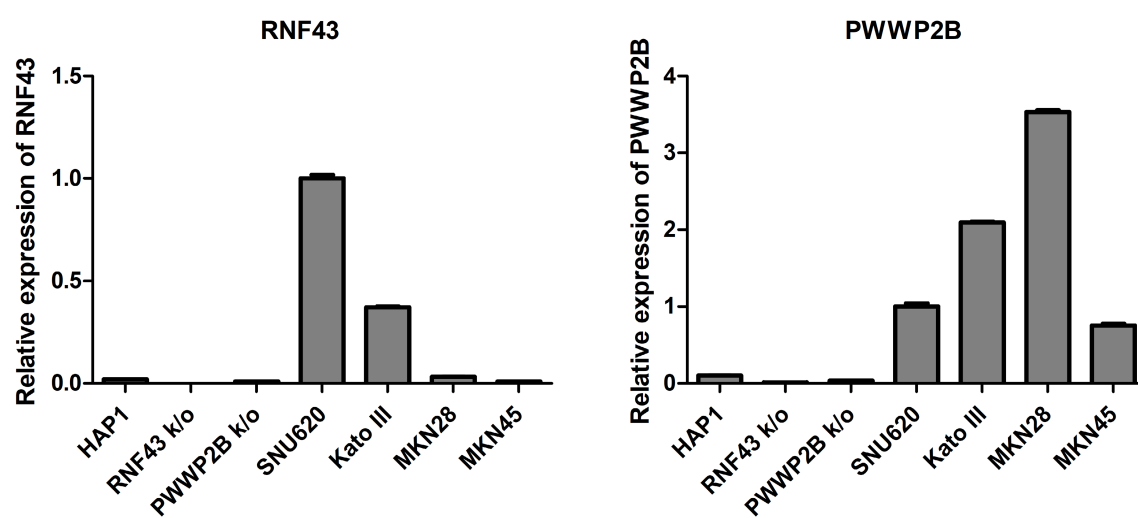

Figure S2.

Supplement: Supplementary file 1 — Supplementary figures. [file jcav12p4616s1.pdf]
